# Murder—Quack Medicines, Etc.

**Published:** 1845-02

**Authors:** 


### MURDER—QUACK MEDICINES, ETC.

The "*Home Journal and Citizen Soldier*," a well conducted weekly newspaper, of the 29th instant, has been sent to us, in which we find a sensible article under the above caption. We are not in the practice of saying much on the subject of quackery, as our readers may have observed, partly because we hope and trust that those who take the *Examiner* are already fully persuaded on that subject, and we would not insult them by supposing otherwise; and because also, as it relates to the victims of such imposture, little that we could say would avail anything against the charge of interest and prejudice alleged by charlatans, just as if they alone were above suspicion of such influences. But we rejoice to see the effort made by others, who stand in a different relation to the community—not that we are prepared to admit that members of our profession are less disinterested, or less sensible of the magnitude of the evil, but that the credulity of the ignorant cannot be so successfully appealed to, by the only available argument against the warning voice of the physician. It is lamentable to read, in some of the newspapers, the atrocious falsehoods and plausible assertions spread before the sick poor, and the ignorant and unsuspecting part of the community, dictated by the basest cupidity, and paid for out of the hard earnings of the destitute. Mechanics who are too lazy to earn their living by honest labour; traders, whose craftiness has overleaped its objects; professed gamblers and thieves;—that such should betake themselves to the despicable traffic is not strange, but it is surprising that respectable publishers can be influenced by the profits of an advertisement to lend their columns to such unholy purposes. Editors and proprietors of newspapers are not ignorant of the character of these advertisers, nor of the baneful influence of their nostrums upon the health of those who take them; and yet the *gilded pill* is swallowed with the same apparent gusto as the wholesome reward afforded by the announcements of legitimate trade and business.

